# AutoSiQ: a curated haploid *Arabidopsis thaliana* inflorescence dataset with a fine-grained silique ontology and a deep learning application for haploid fertility quantification

**DOI:** 10.3389/fpls.2026.1767588

**Published:** 2026-03-18

**Authors:** Liming Zhou, Talukder Z. Jubery, Baskar Ganapathysubramanian, Thomas Lübberstedt, Siddique I. Aboobucker

**Affiliations:** 1 Department of Agronomy, Iowa State University, Ames, IA, United States; 2 Translational AI Research and Education Center, Iowa State University, Ames, IA, United States; 3 Department of Mechanical Engineering, Iowa State University, Ames, IA, United States

**Keywords:** *Arabidopsis thaliana*, dataset, haploid fertility, haploid fertility rate (HFR), image annotation, phenotyping, silique detection, YOLOv5

## Abstract

Doubled haploid (DH) technology can fast-track crop breeding. Haploid induction yields haploids with only one set of genomes, which are usually sterile. Haploid fertility (HF) is the ability of haploid plants to set seed, and it is a critical bottleneck in DH pipelines. Genetic mechanisms to restore HF hold immense potential in DH crop breeding, yet its phenotyping remains manual, destructive, and inconsistent. While recent advances in imaging and machine learning have improved throughput for general plant traits, no curated image dataset exists for *Arabidopsis thaliana* that explicitly represents HF. Here, we present AutoSiQ, a dataset and baseline deep learning pipeline for automated HF quantification. AutoSiQ includes high-resolution scanned inflorescences annotated with a seven-class ontology encompassing green siliques, green fertile siliques, mature siliques, fertile siliques, cracked fertile siliques, cracked siliques, and flowers. This multi-class annotation scheme preserves biologically meaningful information beyond binary fertile/non-fertile distinctions, enabling reliable fertility estimation and future phenotyping applications. We release baseline object detection models (YOLOv5), trained using the AutoSiQ dataset, and evaluate their performance across confidence thresholds. Model predictions strongly correlate with manual counts, achieving R² up to 0.94 for total silique number estimation. We further demonstrate AutoSiQ’s utility for automated haploid fertility rate (HFR) estimation and genotype discrimination between two contrasting genotypes (WT and *bmf2* mutant). A longitudinal analysis identifies ~60 days after sowing (DAS) as the optimal harvest time for maximizing mature silique counts by balancing between the number of immature buds and silique shattering. By releasing both the dataset and baseline code, AutoSiQ provides a reproducible and extensible foundation for high-throughput fertility phenotyping in haploid *Arabidopsis*.

## Introduction

Doubled haploid technology accelerates plant breeding by enabling the rapid production of completely homozygous lines in two generations. Since the first reports of natural haploids in maize (Chase, 1947), DH techniques have been applied to more than 250 plant species (Maluszynski et al., 2003; Li et al., 2025), offering breeders a powerful tool to fix desirable traits efficiently. Significant progress has been made in haploid induction with modern maize inducers possessing induction rates above 10% (Khammona et al., 2025). A key bottleneck remaining in the DH process is haploid fertility (HF) - the ability of haploid plants to set seed through spontaneous or induced chromosome doubling. Conventionally, genome doubling is relied on chemical treatments such as colchicine in maize, which interferes with chromosome segregation during mitosis to restore fertility in haploid plants (Blakeslee and Avery, 1937; Eder and Chalyk, 2002; Boerman et al., 2020). Induced genome doubling remains inefficient, with rates typically ranging from 16% to 49% depending on genotype and method (Melchinger et al., 2016). Colchicine treatment is also toxic and labor-intensive, underscoring the need for alternative strategies. Spontaneous haploid genome doubling has emerged as a promising alternative, with some maize genotypes exhibiting doubling rates exceeding 50% (Boerman et al., 2020). However, maize’s long generation time and cultivation requirements make it less suitable for large-scale genetic studies of HF.

*Arabidopsis thaliana* haploids offer a unique experimental system to study fertility traits because of their small inflorescences, rapid lifecycle, and genetic variation affecting fertility (Ravi and Chan, 2010). Certain accessions (e.g., Col-0, Ler-0, Ws) exhibit measurable HF and genetic mutants in these backgrounds can be leveraged to dissect underlying mechanisms (Aboobucker et al., 2023; Ravi and Chan, 2010). Further, the knowledge generated in *Arabidopsis* provides an opportunity to directly translate the results into crops or at least narrow down the focus, since the fundamental biological process(es) behind HF must be conserved. Therefore, accurate assessment of HF in *Arabidopsis* is essential for identifying fertile haploids and selecting genotypes or treatments that improve fertility. We have developed a method to manually count fertile siliques on inflorescences to estimate HF. However, this process is time-consuming, and laborious. These limitations hinder the scalability and reproducibility of HF phenotyping, particularly in genetic studies involving large plant populations.

Recent advances in plant phenotyping have leveraged imaging and machine learning to automate trait measurement across diverse species and organs. Applications have included leaf morphology, seed traits, stress responses, and reproductive structures (Iyer-Pascuzzi et al., 2010; Wang et al., 2009; Hamidinekoo et al., 2020). In *Arabidopsis*, several computational methods have been proposed for estimating silique number in diploids, including skeletonization-based approaches (Vasseur et al., 2018), graph-tracing algorithms (Augustin et al., 2015), and deep learning models that classify silique parts and reconstruct siliques (Hamidinekoo et al., 2020). However, these methods often target diploid plants and do not explicitly distinguish fertile vs. non-fertile siliques, which is essential for haploid fertility assessment. Moreover, existing silique detection datasets are limited in scope and typically collapse biologically distinct classes into coarse categories, reducing their utility for fine-grained phenotyping or downstream analyses.

Despite this, there is currently no publicly available, curated image dataset that represents haploid inflorescences with explicit fertility annotations. This gap limits the development of standardized, automated pipelines for HF estimation and downstream genotype or treatment comparisons. To address this gap, we developed AutoSiQ, a dataset and baseline deep learning pipeline for automated haploid fertility quantification in *Arabidopsis thaliana*. AutoSiQ comprises high-resolution scanned images of haploid inflorescences annotated with a seven-class silique ontology that captures both fertility status and developmental stage. This ontology goes beyond binary fertile/non-fertile distinctions, preserving biologically meaningful structure that supports accurate fertility estimation and potential future applications.

We pair the dataset with baseline object detection models (YOLOv5) and evaluate their performance for silique detection and counting. We further demonstrate how AutoSiQ enables automated HFR estimation – ratio of fertile siliques to total number of siliques in contrasting genotypes for HF, wild-type (Col-0; WT) and *bmf2* mutant. Finally, we use longitudinal imaging to identify an optimal harvest time (~60 days after sowing) that balances silique maturity and shattering risk, providing a practical guideline for phenotyping.

In summary, this study contributes:

- A curated, fertility-aware dataset of haploid *Arabidopsis* inflorescences with a seven-class ontology
- Baseline detection models for automated silique counting and fertility quantification
- A demonstration of automated HFR estimation and genotype discrimination, and
- A harvest-timing analysis to optimize HF phenotyping.

Together, these contributions establish AutoSiQ as a reproducible and extensible foundation for high-throughput fertility phenotyping in haploid *Arabidopsis thaliana*.

## Materials and methods

### Plant material and growth conditions

The *Arabidopsis thaliana* wild-type (WT) diploid accession used in this study was Columbia (Col-0, stock CS70000) and the *bmf2* (SAIL_303_E05) mutant is as previously described by Komaki and Schnittger (2017). Seeds for both WT and the T-DNA insertion mutant were obtained from the *Arabidopsis* Biological Resource Center (ABRC) at The Ohio State University (Columbus, OH). Haploid seeds were generated by using WT and mutant plants as male donors and crossing them with *cenh3* paternal haploid inducers as female parents (Ravi and Chan, 2010). Putative haploid seeds were separated from aborted seeds using a dissecting microscope (Stereomaster, Fisher Scientific, NY, USA).

To maximize germination efficiency, seeds were surface sterilized and sown onto petri dishes containing Murashige and Skoog (MS) media (Murashige and Skoog, 1962), then stratified at 4°C for 7 days. Following vernalization, plates were transferred to a growth chamber (Percival Scientific, Inc., IA, USA) maintained at 23°C under a 16 h light/8 h dark photoperiod, with light intensity set to approximately 125 µmol m^-^² s^-^¹. After 7 to 10 days, seedlings were transplanted into 6.25 × 6.25 cm^2^ pots filled with Sunshine Professional Growing Mix (Sungro #1 F1P-RS1 [LC1], Sun Gro Horticulture, Agawam, MA, USA) and grown under the same environmental conditions. Diploid WT and mutant seedlings were also grown in parallel to serve as controls during haploid selection. At 20 to 24 days after sowing, haploids were visually identified and separated from hybrids and aneuploids as described by Ravi and Bondada (2016). Only confirmed haploid plants were allowed to continue growing. After 56 days of sowing, watering was halted, and plants were allowed to dry for subsequent haploid fertility evaluation.

### Harvest time determination

To standardize HFR evaluation, we monitored haploid *Arabidopsis* plants from 25 to 65 days after sowing (DAS). Developmental traits - including unopened flower buds, number of branches, visibly mature siliques, and senescing (brown) branches - were recorded at multiple time points. Our goal was to identify a consistent phenotyping window in which mature siliques were abundant, new floral development was minimal, and silique shattering was limited.

**Figure 1:**
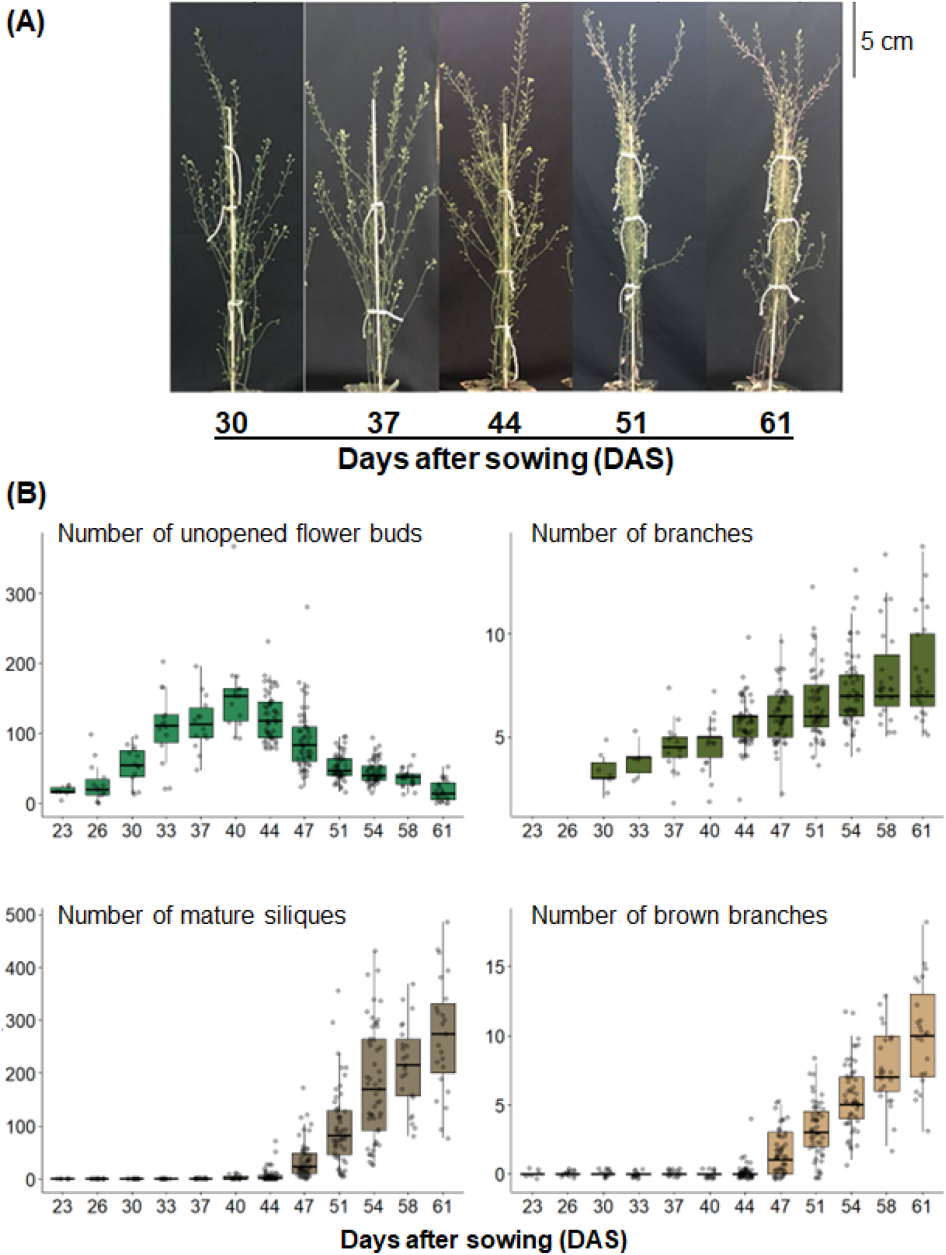
Haploid *Arabidopsis* phenotypic traits observation. **(A)** Representative images of haploid *Arabidopsis* from 30 to 61 days are presented. Scale bar = 5cm. **(B)** Number of unopened flower buds, main branches, mature siliques, and brown branches visible on each haploid *Arabidopsis* were recorded twice a week, and the average number of each trait is expressed with N = 14 to 51 plants. The main branches specifically represent the primary and secondary branches of haploid *Arabidopsis.* The center line in the box plots indicates the median; the bounds of the box refer to the upper (75th percentile) and lower (25th percentile) quartiles, and the whiskers refer to the lowest and highest observations.

As fertile siliques tend to mature early while the apical meristem continues to generate new flowers, it was critical to determine a harvest stage where siliques remained intact and floral noise was minimized. We systematically recorded plant traits at several time points, including total branches, unopened buds, brown siliques, brown branches, and fertile siliques (**Figure 1**). This analysis allowed us to identify an optimal developmental stage (**Figure 1**) at which mature siliques peaked while new flower production declined‐thus enabling consistent and reliable HFR estimation using the AutoSiQ pipeline.

### Manual haploid fertility evaluation

**Figure 2:**
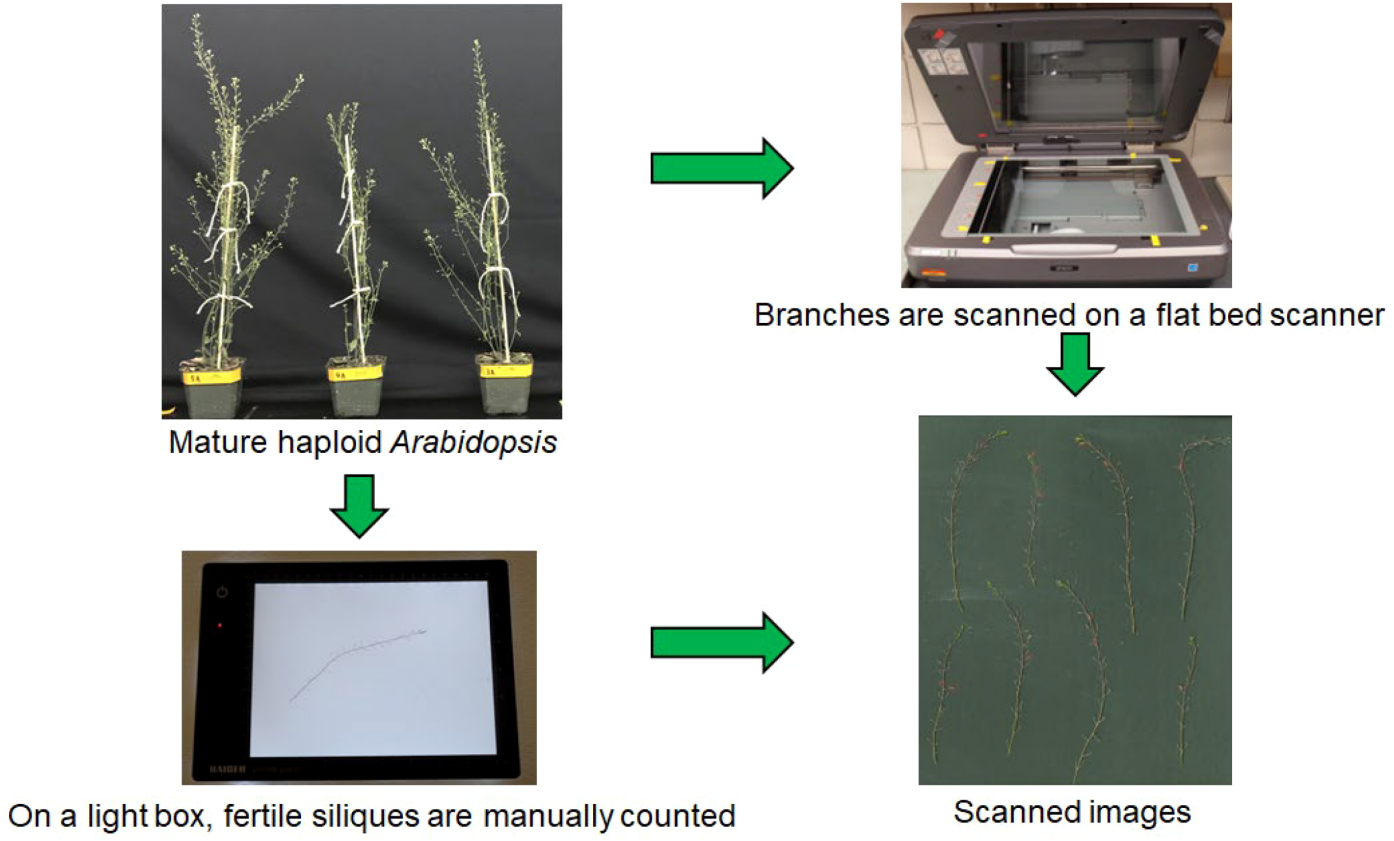
Haploid *Arabidopsis* fertile silique detection and scan acquisition process. Mature *Arabidopsis* haploid plants 61 DAS were used. Branches of the plants were separated from the plant, and fertile siliques were manually counted on a light box and recorded. These same branches were then placed on a flatbed scanner to acquire digital images.

**Figure 3:**
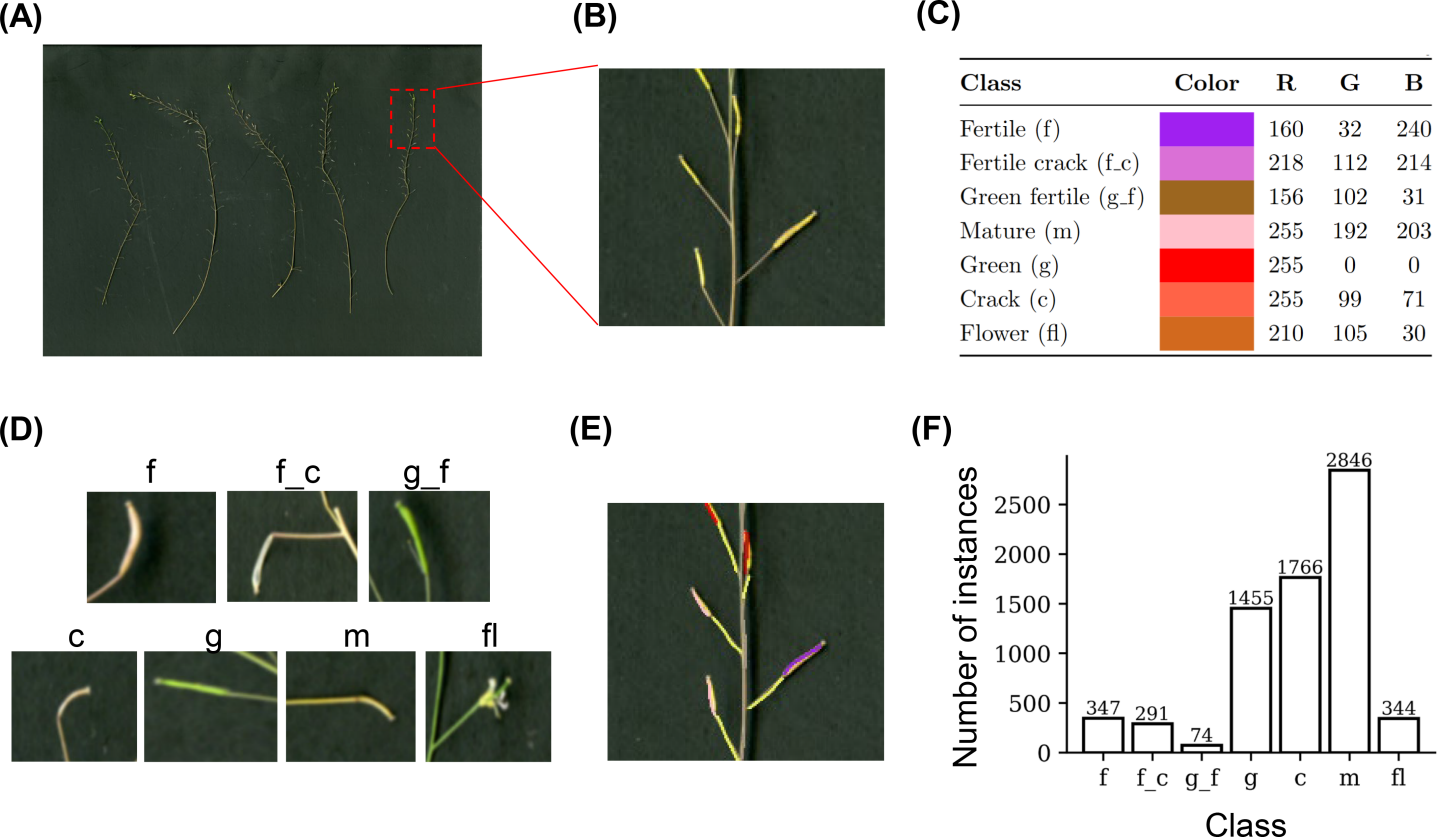
Overview of the AutoSiQ annotation workflow. **(A)** Example scanned RGB image of *Arabidopsis thaliana* inflorescences used as the starting point for annotation. **(B)** A magnified view of a selected region showing individual organs more clearly. **(C)** Color legend indicating the annotation scheme, with RGB values assigned to each organ category: fertile siliques (f), fertile crack (f.c), green fertile (g.f), mature (m), green (g), crack (c), and flower (fl). **(D)** Example cropped image patches corresponding to each annotated class. **(E)** Fully annotated image showing color-coded masks for each organ class. **(F)** Distribution of annotated instances per class across the dataset, highlighting class imbalance.

Shoots were harvested by cutting them at the base. Branches were carefully separated on a bench liner (Fisher Scientific, #14-127-47) to avoid seed loss. Each plant was removed from its pot, and individual branches were arranged on the liner. To aid in visual assessment, a lightbox (Kaiser Slimlite Plano 5000K Battery/AC Lightbox, 8.7 × 6.3”) was used to confirm the presence of fertile siliques (**Figure 2**). A silique was considered fertile if it contained at least one visible seed (**Figure 3D**). The total number of fertile siliques was recorded for each plant to obtain the manual counts.

### Dataset curation and annotation protocol

To generate a curated dataset for automated haploid fertility quantification, we collected *Arabidopsis* inflorescences and scanned them using high-resolution flatbed scanners (HP ENVY Photo 7855 and EPSON Expression 10000 XL; **Figure 2**). Each scan was assigned a unique identifier and paired with its corresponding manual fertile silique count, forming the ground truth foundation of the AutoSiQ dataset.

We curated and annotated a representative subset of these images to build a fine-grained silique ontology specifically designed for haploid fertility phenotyping. Manual annotations were performed by a plant phenotyping expert using Microsoft Paint, with distinct colors assigned to seven biologically meaningful classes: flowers, mature siliques, cracked siliques, green siliques, fertile siliques, cracked fertile siliques, and green fertile siliques (**Figure 3**). A total of 50 high-resolution scanned inflorescence images were manually annotated to generate the AutoSiQ dataset, resulting in several thousand annotated instances distributed across seven silique and floral classes (**Figure 3F**). This ontology goes beyond binary fertile/non-fertile distinctions, enabling downstream models to retain developmental context and fertility information.

**Figure 3** also summarizes class definitions and object frequency distributions. Non-fertile mature flowers (“m”) were the most abundant class, consistent with prior reports that most haploid *Arabidopsis* flowers are sterile (Ravi and Chan, 2010). Cracked siliques (“c”) and green siliques (“g”) were also frequent, followed by fertile siliques (“f”) and cracked fertile siliques (“f_c”) (**Figure 3D**).

To support model training, annotated images were divided into non-overlapping 512 × 512 px patches, and bounding box coordinates and class labels were generated for each patch. The dataset was then split into training (80%) and validation (20%) subsets, establishing a curated, structured dataset for baseline YOLOv5 model development and evaluation. A fixed training-validation split was used to establish a reproducible baseline for future benchmarking on the AutoSiQ dataset; stratified k-fold evaluation may be explored in subsequent studies.

### Baseline model development

#### Architecture

Baseline model for AutoSiQ was developed using the YOLOv5 (You Only Look Once, version 5) object detection framework (Jocher et al., 2021), which provided an optimal balance between accuracy, speed, and deployment efficiency at the time of development. Although more recent models such as YOLOv8, DETR, and DINO have since been introduced, YOLOv5 was state-of-the-art when this work began and was particularly well suited for plant phenotyping tasks in resource-constrained environments.

The YOLOv5 architecture consists of three main components: a backbone (CSPDarknet53; Wang et al., 2019) for multi-scale feature extraction, a neck (PANet; Liu et al., 2018) for cross-scale information fusion, and a detection head (modeled after YOLOv3; Redmon and Farhadi, 2018) for object classification and localization. Each detection layer produces an output tensor of shape *d × (b + n)*, where *d* is the number of anchors, *b* denotes bounding box parameters (x, y, width, height) and objectness score, and *n* is the number of object classes.

AutoSiQ was trained to classify seven biologically relevant object types: green siliques, green fertile siliques, mature siliques, fertile siliques, cracked fertile siliques, cracked siliques, and flowers. Although more recent models such as YOLOv8, DETR, and DINO have since been introduced, YOLOv5 was state-of-the-art when this work began.

#### Training procedure

AutosiQ was trained using a multi-component loss function that included three key terms: (1) a regression loss to optimize bounding box coordinates, (2) binary cross-entropy loss to estimate object presence confidence, and (3) categorical cross-entropy loss for object classification. Weights were initialized using the COCO (Common Objects in Context) dataset, which includes over 330, 000 images and 1.5 million annotated objects across 80 categories. While COCO lacks classes directly relevant to *Arabidopsis*, pretraining facilitated better feature extraction on limited training data.

We employed a two-phase fine-tuning strategy. In the first phase, only the neck and head layers were trained for 10 epochs while the backbone remained frozen. In the second phase, the entire model, including the backbone—was fine-tuned for 900 epochs. During training, samples were shuffled in each epoch to enhance generalization. The AutoSiQ dataset exhibits class imbalance across the seven silique categories (**Figure 3F**). No explicit class-weighted loss or oversampling strategy was used during training. Instead, robustness to imbalance was partially achieved through YOLOv5’s objectness-aware loss formulation, in which classification and localization losses are conditioned on the predicted presence of an object, and through conservative geometric and photometric data augmentation. For downstream haploid fertility estimation, fertile-related subclasses were aggregated into a binary fertile vs. non-fertile distinction, reducing sensitivity to phenotypic imbalance.

#### Data augmentation

To prevent overfitting and improve robustness, both geometric and photometric augmentations were applied during training. Geometric transformations included random scaling, translation, cropping, rotation, and horizontal flipping. We also evaluated advanced composition-based augmentations such as CutMix and Mosaic. CutMix involved blending patches from two images, while Mosaic combined four images into one to simulate spatial variability. These approaches, however, did not improve performance for scanned inflorescence images and were therefore not used in the final training configuration. We attribute this to the fact that siliques exhibit highly similar local appearance, and fertility classes are primarily distinguished by global completeness rather than local texture, making partial recombination or truncation detrimental.

Photometric augmentations adjusted image hue, saturation, and brightness to account for differences in lighting and sensor conditions. These augmentations introduced diversity in object appearance and context, enhancing the model’s ability to generalize to unseen samples.

##### Confidence threshold optimization

Model performance was evaluated using two standard object detection metrics: mean average precision (mAP) and F1-score.

mAP measures the mean of average precision across all object classes by calculating the area under the precision-recall curve:


$$AP=\int_{0}^{1}p(r)dr$$

Where p(r) is the precision as a function of recall r. This metric reflects both the accuracy and completeness of detection across different confidence thresholds. In addition, we computed the F1-score, which is the harmonic means of precision and recall. This metric is particularly useful when dealing with class imbalance, as it provides a balanced evaluation of both false positives and false negatives. The F1-score is defined as:


F1=2P*R/(P+R)

Where P and R are the precision and recall.


P=TP/(TP+FP)

R=TP/(TP+FN)

Here, TP, FP, and FN represent true positives, false positives, and false negatives, respectively.

To identify the optimal confidence threshold, we employed a genetic algorithm to explore hyperparameter combinations that maximized mAP. This approach enabled effective model tuning under class imbalance and diverse object characteristics.

##### Software and implementation details

Object detection was performed using the Ultralytics YOLO framework (v8.3.228) via the yolo command-line interface, using the YOLOv5 small model (yolov5s.pt). All experiments were conducted in a Conda environment using Python 3.8.20 (conda-forge), PyTorch 2.4.1+cu124, and CUDA 12.4.

#### Phenotypic trait quantification and validation

AutoSiQ outputs included object counts for all seven silique and flower categories. To estimate haploid fertility, we summed the number of fertile siliques, cracked fertile siliques, and cracked siliques. Model predictions were compared against manual counts using Pearson correlation (R²), with particular focus on fertile silique detection accuracy for HFR quantification.

## Results

### Optimal harvest window for haploid fertility evaluation

*Arabidopsis* has an indeterminate growth phenotype - new flowers are produced simultaneously while old flowers mature and their siliques shatter. This is more pronounced in haploids, since they produce flowers profusely (Ravi and Chan, 2010). Therefore, it is essential to identify an optimal window - for harvesting *Arabidopsis* plants to estimate HF - to balance between the number of new flowers and shattering siliques. To this end, various growth traits were recorded from *Arabidopsis* haploids (**Figure 1**). Flower buds began appearing 25 days after sowing and peaked around day 40 before declining (**Figure 1**). Correspondingly, mature siliques first appeared at approximately 40 days - about 14 days after flower bud emergence - consistent with silique maturation timelines in diploid *Arabidopsis* (Boyes et al., 2001). The number of mature siliques reached its maximum at 61 days, while flower bud counts dropped to their lowest at this stage (**Figure 1**). The number of main branches plateaued at six or seven by day 40, and brown branches began appearing around this time. Notably, the increase in brown branches positively correlated with the accumulation of mature siliques (**Figure 1**). Taken together, these observations suggest that 61 days after sowing is the optimal time point to harvest *Arabidopsis* for haploid fertility (HF) evaluation. To our knowledge, this is the first report of a detailed characterization of *Arabidopsis* haploid reproductive growth.

### Baseline object detection performance

**Figure 4:**
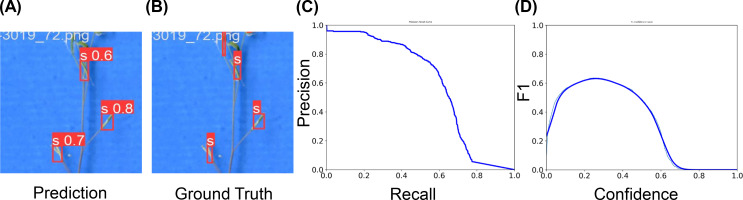
Model performance for total silique detection. **(A)** Example prediction showing detected siliques with corresponding confidence scores. **(B)** Ground truth annotation for the same sample. **(C)** Precision–Recall curve illustrating model performance across varying recall levels. The area under the curve corresponds to the Average Precision (AP) at IoU 0.5. **(D)** F1–Confidence curve showing the relationship between confidence threshold and F1-score. The peak of the curve indicates the optimal threshold used for evaluation.

**Figure 5:**
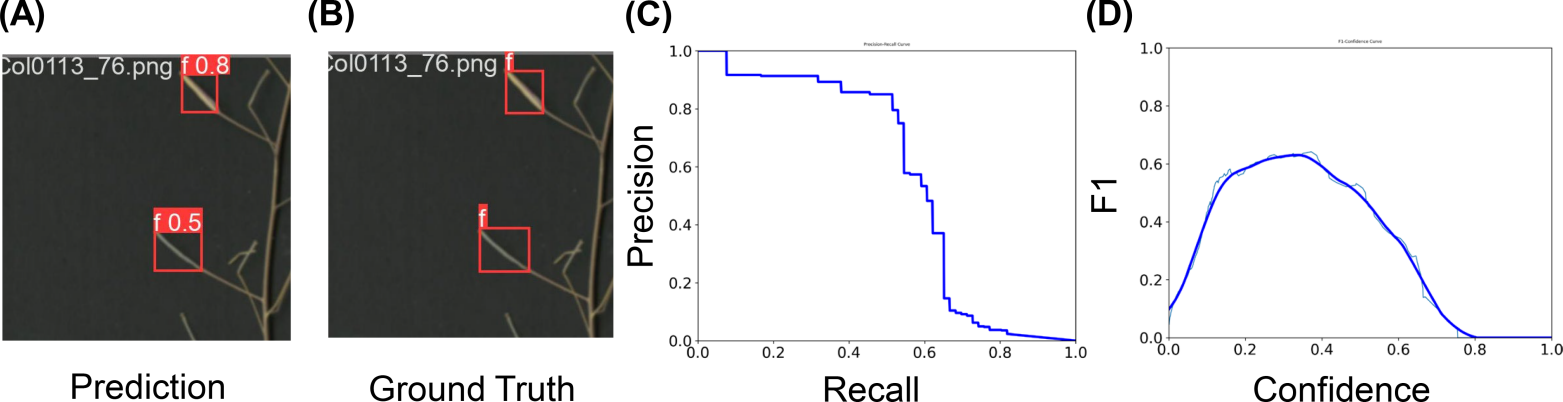
Model performance for fertile silique detection. **(A)** Example prediction showing detected fertile siliques with corresponding confidence scores. **(B)** Ground truth annotation for the same sample. **(C)** Precision–Recall curve illustrating model performance across varying recall levels. The area under the curve corresponds to the Average Precision (AP) at IoU 0.5. **(D)** F1–Confidence curve showing the relationship between confidence threshold and F1-score. The peak of the curve indicates the optimal threshold used for evaluation.

The YOLOv5 model trained on the AutoSiQ dataset showed strong detection performance for both total and fertile silique detection tasks (**Figures 4**, **5**).

**Figure 6:**
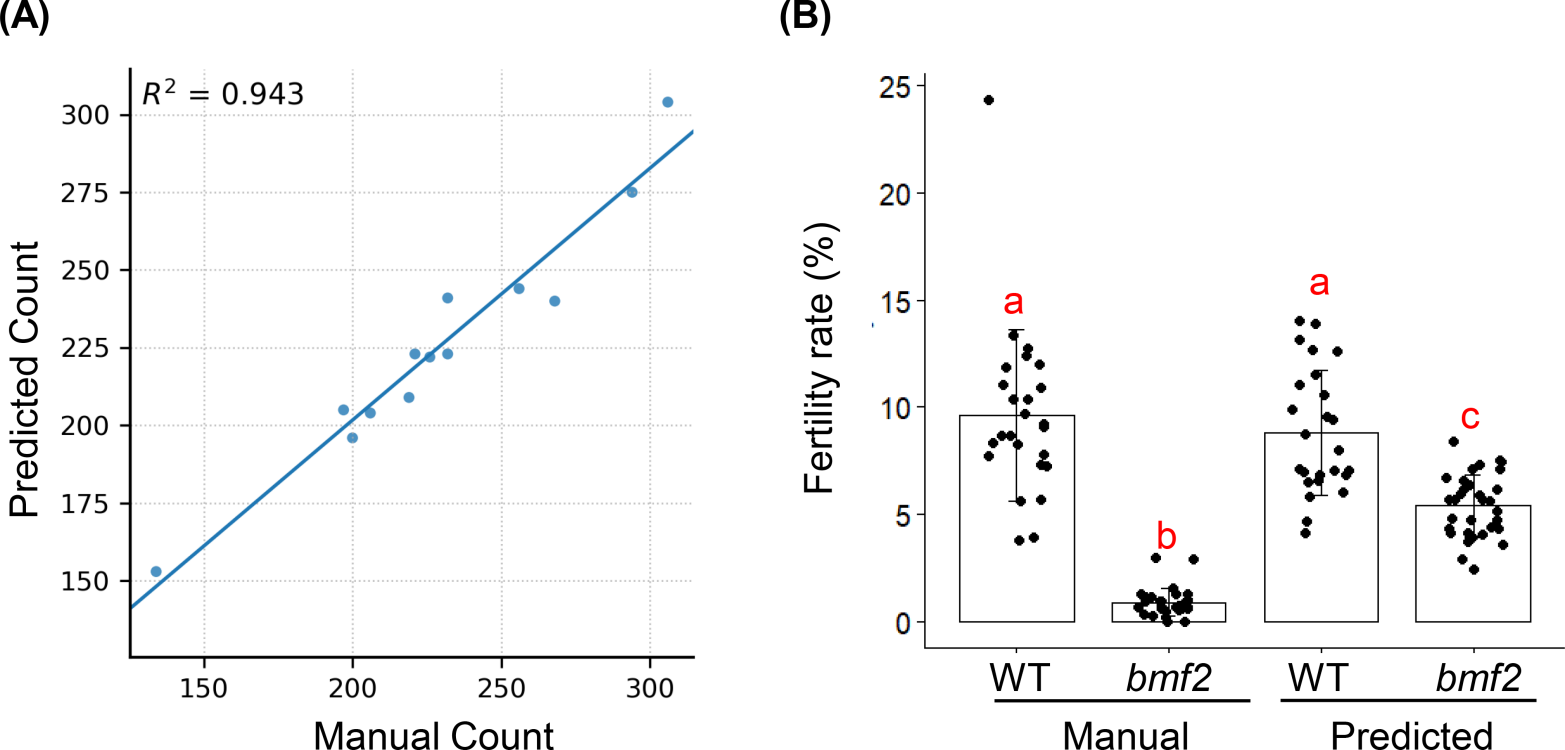
Validation of AutoSiQ silique counts and fertility rates. **(A)** Scatter plot comparing automated total silique counts from AutoSiQ with manual ground truth counts across all test images (confidence threshold = 0.6). Each dot represents one inflorescence; a straight line indicates a linear fit (R² = 0.94). **(B)** Comparison of fertility rate (HFR) estimation by manual and AutoSiQ method in WT (N = 29 plants) and *bmf2* (N = 32 plants) genotypes, showing genotype-specific difference. One-way ANOVA test was conducted followed by Tukey test at a 95% confidence interval. Different letters show statistical signiicance.

For total silique detection, the model achieved an overall mAP@0.5 of 60%, with consistently high precision across most recall levels (**Figure 4**). The precision–recall curve indicates that the model maintained a precision above 0.8 over a wide recall range, with performance gradually decreasing at higher recall. The F1-confidence curve (**Figure 4**) peaked at approximately 0.63 around a confidence threshold of 0.26, suggesting this as the optimal threshold for evaluation. Qualitative examples comparing predicted and ground truth bounding boxes are shown in **Figure 4**. These results highlight the model’s effectiveness in identifying total siliques across diverse samples and conditions. To assess the accuracy of AutoSiQ for estimating silique counts, we compared automated predictions with manual ground truth counts across all samples. For total silique counts, AutoSiQ showed a strong linear relationship with manual counts (R² = 0.94; **Figure 6A**), indicating a high degree of concordance between the automated pipeline and human annotation. This result demonstrates that AutoSiQ can reliably quantify total siliques across diverse inflorescences.

For fertile silique detection, the model achieved strong single-class performance, with a mAP@0.5 of 56%. At the optimal confidence threshold (≈ 0.34), the model reached a precision of 0.80, recall of 0.45, and a peak F1 score of 0.63 (**Figure 5**). This high precision–recall balance indicates the model’s reliability in capturing fertile siliques with minimal false positives. Accurate fertile silique detection provides a solid foundation for downstream fertility estimation, enabling phenotypic analysis of reproductive success at the inflorescence scale.

### Validation with genotypes of contrasting fertility

We further validated the biological relevance of the automated fertility estimates using two *Arabidopsis* genotypes with contrasting haploid fertility: WT and *bmf2* (reduced haploid fertility mutant; to be published elsewhere). With the ability to estimate total silique counts, it is possible to estimate HFR using total and fertile silique counts. Manual counting confirmed a significant reduction in HFR in *bmf2* compared to WT (**Figure 6B**). Importantly, the AutoSiQ pipeline reproduced this genotype-specific difference using automated counts (**Figure 6B**), demonstrating that the model captures biologically meaningful fertility differences. These results support the use of AutoSiQ for high-throughput, objective fertility phenotyping in genetic studies.

## Discussion

Doubled haploid (DH) technology has the potential to greatly accelerate crop breeding. However, one of the key bottlenecks for its widespread application is genome doubling of haploids, which are typically male sterile. In maize, for example, genome doubling is commonly achieved through colchicine treatment followed by manual transplanting of treated seedlings (Boerman et al., 2020). Identifying genetic mechanisms to overcome haploid male fertility (HMF) is therefore of high significance. *Arabidopsis thaliana*, as a model species, exhibits some degree of haploid fertility (HF) (Ravi and Chan, 2010). Additionally, the availability of mutant libraries (O’Malley et al., 2015) presents an opportunity to identify genes regulating HF at a large scale. To support this effort, we developed AutoSiQ - a high-throughput image analysis pipeline designed to evaluate HF in *Arabidopsis* (**Figures 2**, **3**).

Determining the optimal harvest time for evaluating HF in haploid *Arabidopsis* is critical. Owing to its indeterminate growth habit, *Arabidopsis* simultaneously produces new flowers while older flowers mature and shatter. This is particularly pronounced in haploids, where profuse flowering occurs due to their largely sterile nature (Ravi and Chan, 2010). Our analysis across developmental stages revealed that flower production peaked at 48 days and declined significantly by 61 days after sowing (**Figure 1**). Concurrently, most siliques have reached maturity by this time (**Figure 1**), indicating that day 61 (late in week 9) represents a balanced stage - where cracked siliques are maximized, and few new buds are being initiated.

The bud count in **Figure 1B** reflects unopened flower buds, which will ultimately develop into mature siliques. Notably, most fertile siliques were located at the top of each main branch–whether primary or secondary - suggesting that fertile siliques originate primarily from buds produced during weeks 7 to 8. If plants were harvested during week 7, the ongoing bud production could lead to an underestimation of the final HFR. By late week 8, the bud count enters a stationary phase, indicating that harvesting at day 61 would not significantly affect total silique number. However, delaying harvest beyond this point could lead to increased silique shattering. Thus, day 61 after sowing represents the optimal harvest window for reliable HF assessment in haploid *Arabidopsis*. To our knowledge, this is the first report of a detailed characterization of *Arabidopsis* haploid reproductive growth.

According to growth stage definitions by Boyes et al. (2001), diploid *Arabidopsis* inflorescences should be harvested 2–4 days after stage 6.90, when the number of fertile siliques peaks. Our results show that haploids deviate from this timeline - likely due to their profuse and extended flower production - highlighting the need for haploid-specific harvest guidelines. Identifying this optimal window (day 61) has proven valuable for capturing HF phenotypes across different genotypes, as demonstrated in **Figure 6**. It should, however, be noted that the optimal harvest time for HF assessment might differ when using other ecotypes that are not tested here. A pilot study to assess optimal harvest time using the standards here is suggested for future work with various ecotypes.

This study presents AutoSiQ, a dataset and baseline deep learning pipeline for automated HF quantification. To our knowledge, this is the first report of its kind in the field. AutoSiQ includes high-resolution scanned inflorescences annotated with a seven-class ontology encompassing green siliques, green fertile siliques, mature siliques, fertile siliques, cracked fertile siliques, cracked siliques, and flowers (**Figure 3**). This fine-grained annotation scheme preserves biologically meaningful information beyond binary fertile/non-fertile distinctions, enabling reliable fertility estimation and future phenotyping applications. The baseline object detection models (YOLOv5) presented in this study were trained using the AutoSiQ dataset and evaluated for their performance across confidence thresholds (**Figures 4**, **5**). Model predictions strongly correlate with manual counts, achieving R² up to 0.94 for total silique number estimation. We further demonstrate AutoSiQ’s utility for automated HFR estimation in two contrasting genotypes (**Figure 6**).

In addition to quantifying fertile siliques, total silique counts could also be estimated using AutoSiQ. This gives the ability to estimate HFR - defined as the ratio of fertile siliques to total siliques - which is a more robust and informative trait for comparing HF across genotypes and other ecotypes (**Figure 6**). However, manual counting of total and fertile siliques is extremely time-consuming and often impractical, particularly for large-scale screens. In this context, automated image analysis tools offer a transformative advantage. Previous studies have highlighted the utility of image-based phenotyping to extract multiple physiological traits in plants (Iyer-Pascuzzi et al., 2010; Wang et al., 2009). For both high-throughput screening and detailed genetic studies, the need for accurate, multi-trait phenotypic measurements is growing. Given the high correlation between AutoSiQ predictions and ground truth values, our tool provides a promising approach for rapid and consistent HF evaluation in haploid *Arabidopsis.* Although AutoSiQ slightly overestimated fertility rates for the *bmf2* mutant compared to manual scoring, the predicted values still preserved the expected biological contrast, with *bmf2* remaining significantly lower than WT (**Figure 6B**). The overestimation of HFR observed for the *bmf2* mutant (**Figure 6B**) could be primarily attributed to the limited representation of fertile siliques for this mutant in the training dataset. The relatively small number of fertile samples introduce class imbalance and may reduce model generalization for mutant-specific fertility patterns. Future work may mitigate this effect through increased sampling of mutant-specific phenotypes and improved class balance during training.

Together, these results highlight the utility of automated, high-throughput phenotyping for both large-scale screens and detailed genetic studies aimed at understanding and enhancing haploid fertility. Although YOLOv5 was a suitable choice when AutoSiQ was developed, we acknowledge the potential of newer object detection architectures - including YOLOv8, DETR, and DINO - that offer improved accuracy, robustness, and architectural innovations.

### Limitations and future directions

A primary limitation of the current AutoSiQ pipeline is its reliance on high-resolution scans of excised inflorescence branches acquired under controlled conditions. While this setup enables precise annotation and high detection accuracy, it requires destructive sampling and limits direct applicability to *in situ* or field-based phenotyping. In addition, flatbed scanning minimizes background clutter and perspective variation, conditions that may not be representative of whole-plant or field imagery. Nevertheless, the modular design of AutoSiQ makes it amenable to extension beyond scanned branches. With appropriate training data, the detection model could be adapted to whole-plant images or field-acquired data by incorporating multi-scale detection, more diverse backgrounds, and varying viewpoints. Such extensions would enable non-destructive, *in situ* fertility assessment and broaden the applicability of AutoSiQ to larger-scale phenotyping platforms and crop systems.

## Conclusion

In this study, we introduced AutoSiQ, a high-throughput, image-based analysis pipeline specifically designed for the automated quantification of silique fertility traits in haploid *Arabidopsis thaliana*. By combining state-of-the-art object detection with image-based phenotyping, AutoSiQ provides a scalable, reproducible, and cost-effective framework for estimating HFR - a critical trait in genetic studies and doubled haploid (DH) breeding programs. The AutoSiQ model was developed using the YOLOv5 object detection framework, selected for its optimal trade-off between accuracy, inference speed, and ease of deployment at the time of development. The model was trained to detect and classify seven biologically relevant floral structures from high-resolution scanned inflorescence images. Evaluation against manually annotated ground truth demonstrated a strong correlation, particularly for fertile and cracked fertile siliques—key indicators of haploid fertility. These results confirm the model’s utility for high-confidence trait extraction.

Although YOLOv5 was a suitable choice when AutoSiQ was developed, we acknowledge the potential of newer object detection architectures**-**including YOLOv8, DETR, and DINO**-**that offer improved accuracy, robustness, and architectural innovations. Future iterations of AutoSiQ may benefit from benchmarking and integrating these advanced models.

To facilitate reproducibility and encourage further research, we are releasing the fully annotated dataset used in this study as an open-source resource. This enables researchers to fine-tune or test novel detection architectures, adapt the pipeline to other plant species or floral structures, and contribute to the growing landscape of automated plant phenotyping.

In conclusion, AutoSiQ provides a robust and extensible solution for rapid, objective, and large-scale quantification of fertility traits in *Arabidopsis thaliana*. By addressing key bottlenecks in phenotyping, this tool advances the potential for deeper genetic understanding and enhanced DH breeding efficiency, with implications for broader crop improvement initiatives.

## Data Availability

The datasets presented in this study can be found in online repositories. The names of the repository/repositories and accession number(s) can be found below: https://zenodo.org/records/17905566.
